# Evaluation der multimodalen perioperativen Versorgung mittels Patienten-App in der kolorektalen Chirurgie – erste klinische Erfahrungen und Patientenzufriedenheit

**DOI:** 10.1055/a-2862-3085

**Published:** 2026-06-08

**Authors:** Mona Breßer, Charlotte Wiedemann, Nils Sommer, Ambra Marx, Paula Tups, Hannah Schulz, Jörg C. Kalff, Tim Vilz, Jonas Dohmen

**Affiliations:** 1 Klinik und Poliklinik für Allgemein-, Viszeral-, Gefäß- und Transplantationschirurgie 39062 Universitätsklinikum BonnBonnNRWDeutschland; 2 Bonn Surgical Technology Center (BOSTER) 9374 Universität BonnBonnNRWDeutschland; 3 Psychosomatische Medizin und Psychotherapie 39062 Universitätsklinikum BonnBonnNRWDeutschland; 4 Onkologische Ernährungsberatung, Abteilung für Integrierte Onkologie, Centrum für Integrierte Onkologie (CIO) Bonn 39062 Universitätsklinikum BonnBonnNRWDeutschland

**Keywords:** Patienten-App, digitale Chirurgie, Prähabilitation, digitale Gesundheitsanwendungen, Enhanced Recovery after Surgery (ERAS), Fast-Track-Chirurgie, digital health applications, fast-track surgery, prehabilitation, mobile health (mHealth), enhanced recovery after surgery (ERAS), perioperative care

## Abstract

**Hintergrund:**

Mobile Gesundheitsanwendungen (mHealth) gewinnen in der Chirurgie zunehmend an Bedeutung, insbesondere zur Unterstützung der Prähabilitation, perioperativen Patientenführung und poststationären Überwachung. Im deutschsprachigen Raum liegen bislang jedoch nur begrenzte Daten zur systematischen Integration solcher Anwendungen in den chirurgischen Versorgungsalltag vor. Ziel dieser Pilotstudie war es, die Machbarkeit, Nutzerakzeptanz und Zufriedenheit einer neu entwickelten multimodalen perioperativen Patienten-App in der Kolorektalchirurgie zu evaluieren.

**Patienten:**

In diese prospektive Beobachtungsstudie wurden 14 Patientinnen und Patienten eingeschlossen, die sich zwischen Juni und Oktober 2025 einer elektiven kolorektalen Operation unterzogen und perioperativ eine mPOM-basierte (multimodales perioperatives Management) Patienten-App nutzten. Die Anwendung umfasste prähabilitative Module, edukative Inhalte zur stationären Phase sowie ein strukturiertes poststationäres Monitoring mit standardisierten Symptomabfragen und Eskalationshinweisen. Die Evaluation erfolgte 30 Tage postoperativ mittels des validierten GER-MAUQ Fragebogens sowie projektspezifischer Fragen.

**Ergebnisse:**

Alle Teilnehmenden nutzten die App regelmäßig bis zum Abschluss der Behandlung. Benutzerfreundlichkeit, Verständlichkeit der Inhalte und Navigation wurden überwiegend sehr positiv bewertet. Sämtliche Patientinnen und Patienten gaben an, durch die App in der präoperativen Vorbereitung unterstützt worden zu sein; 85% berichteten eine erleichterte postoperative Erholung. Rund 80% fühlten sich sowohl peri- als auch postoperativ weniger alleingelassen. Erinnerungsfunktionen, die digitale Erfassung klinisch relevanter Parameter sowie der dauerhafte Zugriff auf Gesundheitsinformationen erhielten besonders hohe Bewertungen. Zudem gaben 71% der Befragten an, dass der Zugang zu Gesundheitsleistungen durch die App erleichtert worden sei. Die Gesamtzufriedenheit sowie die Weiterempfehlungsbereitschaft waren insgesamt hoch.

**Schlussfolgerung:**

Die Ergebnisse dieser Pilotstudie zeigen, dass eine multimodale digitale Patientenführung im gesamten perioperativen Verlauf der Kolorektalchirurgie gut implementierbar ist und hohe Akzeptanz findet. Digitale Anwendungen können die Prähabilitation wirksam unterstützen, das subjektive Sicherheitsempfinden stärken, das Selbstmanagement fördern und organisatorische Prozesse entlasten. Zur belastbaren Beurteilung klinischer Outcome-Parameter sowie gesundheitsökonomischer Effekte sind größere prospektive Studien erforderlich.

## Abstract (en)

**Background:**

Mobile health (mHealth) applications are gaining increasing importance in surgery, particularly for supporting prehabilitation, structured perioperative patient guidance, and post-discharge monitoring. However, in German-speaking countries, data on the systematic integration of such applications into routine surgical care remain limited. The aim of this pilot study was to evaluate the feasibility, user acceptance, and satisfaction associated with a newly developed multimodal perioperative patient app in colorectal surgery.

**Methods:**

This prospective observational study included 14 patients who underwent elective colorectal surgery between June and October 2025 and used an mPOM-based (multimodal perioperative management) patient app throughout the perioperative period. The application comprised prehabilitation modules, educational content for the inpatient phase, and structured post-discharge monitoring with standardised symptom assessments and predefined escalation criteria. Evaluation took place 30 days postoperatively using the validated GER-MAUQ questionnaire and additional project-specific items.

**Results:**

All participants used the app regularly until completion of treatment. Usability, clarity of the content, and navigation were rated very positively. All patients reported that the app supported their preoperative preparation; 85% also perceived their postoperative recovery as facilitated. Approximately 80% felt less left alone during both the perioperative and post-discharge phases. Reminder functions, digital recording of clinically relevant parameters, and continuous access to health information received particularly high ratings. In addition, 71% of respondents stated that the app facilitated access to healthcare services. Overall satisfaction and willingness to recommend the app were high.

**Conclusion:**

The results of this pilot study demonstrate that multimodal digital patient guidance throughout the entire perioperative pathway in colorectal surgery is feasible and well accepted. Digital applications can effectively support prehabilitation, enhance patients’ perceived sense of safety, promote self-management, and relieve organisational processes. However, larger prospective studies are required to reliably assess clinical outcome parameters and health economic effects.

## Einleitung

Mobile Patienten-Apps (mHealth apps) werden in der Medizin zunehmend als ergänzendes Instrument zur strukturierten Patientenbetreuung eingesetzt 1 2 3 . In der Chirurgie ermöglichen sie insbesondere die Unterstützung der Prähabilitation 4 5 6 7 8 , die standardisierte Bereitstellung von Informationen sowie die Erhebung patientenberichteter Daten. Darüber hinaus können sie zur poststationären Überwachung beitragen und damit Elemente eines „Hospital-at-Home“-Ansatzes abbilden 9 10 11 . Digitale Anwendungen bieten somit das Potenzial, die gesamte perioperative Patientenreise in einem integrierten System abzubilden.

Gerade in der Viszeralchirurgie ergeben sich daraus relevante Chancen: Die Inhalte moderner perioperativer Konzepte wie Enhanced Recovery After Surgery (ERAS) bzw. multimodales perioperatives Management (mPOM) lassen sich digital strukturiert vermitteln, patientenindividuell ausspielen und im Verlauf überwachen 12 13 14 15 16 . Hierdurch können Parameter wie Länge des Krankenhausaufenthalts oder Wiederaufnahmerate positiv beeinflusst werden 9 10 . Gleichzeitig könnte die in vielen Studien als verbesserungswürdig beschriebene Adhärenz 17 18 , insbesondere bei patientengesteuerten Maßnahmen wie Nikotin- oder Alkoholabstinenz sowie der frühzeitigen postoperativen Mobilisation, durch digitale Unterstützung nachhaltig verbessert werden.

Während in Ländern wie den Niederlanden bereits umfangreiche Erfahrungen mit chirurgischen Patienten-Apps vorliegen 7 13 15 19 20 , ist deren Einsatz im deutschsprachigen Raum bisher kaum etabliert.

Vor diesem Hintergrund wurde am Universitätsklinikum Bonn in Kooperation mit der Firma Luscii Healthtech B. V. erstmals eine umfassende Patienten-App für kolorektalchirurgische Eingriffe entwickelt. Die Anwendung bildet sämtliche Abschnitte der perioperativen Patientenreise ab – von der präoperativen Prähabilitation über die stationäre Behandlung bis zur poststationären Nachsorge – und basiert auf den Prinzipien des mPOM-Konzeptes, wie sie in der S3-Leitlinie „Perioperatives Management bei gastrointestinalen Erkrankungen (POMGAT)“ definiert sind 21 . Die Kolorektalchirurgie eignet sich hierfür besonders, da Fast-Track- und mPOM-Protokolle in diesem Fachgebiet bereits gut validiert und klinisch breit implementiert sind 22 23 24 .

Ziel dieser prospektiven Beobachtungsstudie war es, erste klinische Erfahrungen mit der App im Routinebetrieb zu beschreiben und die Nutzerakzeptanz sowie Zufriedenheit der initialen Pilotkohorte zu evaluieren. Hierfür wurden die Rückmeldungen der ersten 14 Patienten und Patientinnen analysiert, die das Programm vollständig durchlaufen haben.

## Patienten und Methoden

### Studienpopulation und Setting

Die vorliegende Arbeit wurde als prospektive Pilotstudie durchgeführt. Eingeschlossen wurden 14 Patientinnen und Patienten, die sich zwischen Juni und Oktober 2025 einer elektiven kolorektalen Operation unterzogen und perioperativ die App nutzten. Die Dauer der präoperativen Nutzungsphase der App variierte abhängig vom individuellen Zeitpunkt der Operationsplanung. In der Regel erfolgte die Aktivierung der App nach der Operationsindikation im Rahmen der präoperativen Vorbereitung und lag im Bereich von wenigen Tagen bis wenigen Wochen vor dem geplanten Eingriff. Von 19 angesprochenen Personen stimmten 14 (74%) der Teilnahme zu; Nichtteilnahmen erfolgten ausschließlich aufgrund fehlender digitaler Affinität.

### Intervention: digitale Patienten-App

**Abb. 1:**
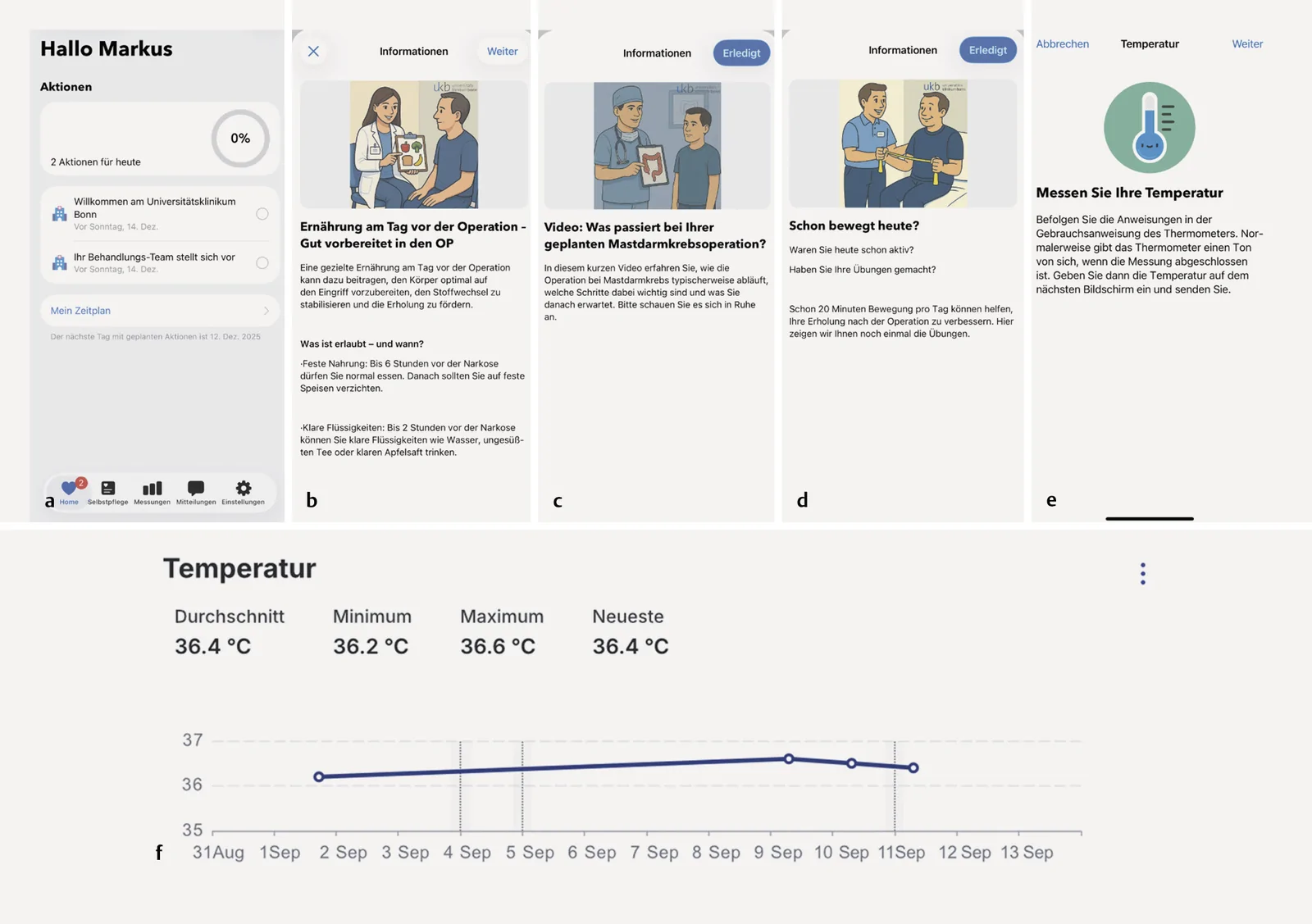
Übersicht über die Funktionen der Patienten-App anhand ausgewählter Anwendungsbildschirme. **a** Startbildschirm mit Darstellung des individualisierten Therapiefortschritts. **b** Edukativer Textbaustein zur präoperativen Ernährung. **c** Videobasierte Erstinformation zum operativen Eingriff. **d** Erinnerungsfunktion exemplarisch dargestellt anhand der Mobilisationsaufforderung. **e** Systematische Symptomabfrage exemplarisch am Beispiel der Temperaturmessung. **f** Für das medizinische Fachpersonal einsehbares Dashboard mit Visualisierung exemplarischer Temperaturverläufe. Quelle: Luscii Healthtech B. V., Programm Peri-OP Kolorektal und Universitätsklinikum Bonn, Klinik und Poliklinik für Allgemein-, Viszeral-, Gefäß- und Transplantationschirurgie.

Es wurde eine digitale Patienten-App eingesetzt, die auf einer standardisierten perioperativen Begleitung nach den Prinzipien des mPOM-Konzeptes auf Grundlage der POMGAT S3-Leitlinie 21 24 basierte. Die App diente sowohl der Informationsvermittlung als auch der systematischen Erfassung patientenseitig eingegebener Daten. Sie kombinierte hierfür strukturierte Lerninhalte, Push-Benachrichtigungen sowie standardisierte Skalen zur Eingabe patientenseitiger Informationen. Eine dauerhaft verfügbare digitale Wissensbibliothek stellte alle edukativen Inhalte bereit, während ein individueller Fortschrittsmonitor den Patientinnen und Patienten ihren jeweiligen Bearbeitungsstand anzeigte ( Abb. 1 **a** ). Die App war in prästationäre, stationäre und poststationäre Anwendungsphasen gegliedert.

Prästationär standen prähabilitative Module zu körperlicher Aktivität und Physiotherapie, psychoonkologischer Unterstützung einschließlich angeleiteter Entspannungsübungen wie Bodyscan und Progressive Muskelrelaxation, zur Nikotin- und Alkoholkarenz sowie zur praxisnahen Ernährungsoptimierung mit konkreter Rezeptempfehlungen zur Verfügung ( Abb. 1 **b** ). Die Inhalte orientierten sich an zentralen Prinzipien moderner mPOM-Konzepte, insbesondere an präoperativer Patientenedukation, Prähabilitation und Optimierung von Ernährungsstatus und Risikofaktoren. Ergänzend wurden organisatorische Inhalte bereitgestellt (z. B. Packlisten, Wegbeschreibungen). Die Wissensvermittlung erfolgte textbasiert und mittels Videomaterial, u. a. zu physiotherapeutischen Übungen zu Hause oder zur Operationsaufklärung ( Abb. 1 **c** ). Darüber hinaus wurde die präoperative Gewichtsentwicklung dokumentiert, um unbeabsichtigte Gewichtsverluste als potenzielles Risikozeichen frühzeitig zu erkennen. Aufgrund der im klinischen Alltag häufig relativ kurzen Zeitspanne zwischen Operationsplanung und Eingriff war eine längere Verlaufsbeobachtung in der vorliegenden Pilotkohorte jedoch nur eingeschränkt möglich.

Während des stationären Aufenthalts wurden strukturierte Informationen zum Behandlungsverlauf bereitgestellt, u. a. zu modernen Nüchternheitsregeln und selektiver Darmvorbereitung, minimalinvasiven Operationsverfahren sowie multimodalen Analgesiekonzepten als zentrale Elemente moderner ERAS-/mPOM-Protokolle. Ergänzend erhielten die Patientinnen und Patienten digitale Erinnerungen zu mPOM-relevanten Maßnahmen wie Frühmobilisation, Atem- und Entspannungsübungen sowie frühzeitiger oraler Ernährung um die aktive Mitwirkung an zentralen Elementen der perioperativen Behandlung zu unterstützen ( Abb. 1 **d** ).

Im poststationären Verlauf erfolgte ein systematisches Monitoring per standardisierter Symptomabfragen (z. B. Wundsituation, Schmerzintensität, Körpertemperatur; Abb. 1 **e** ). Ergänzend wurden definierte Eskalationshinweise integriert, die den Patientinnen und Patienten bei auffälligen Eingaben – etwa Fieber oder zunehmenden Schmerzen – eine Empfehlung zur zeitnahen ärztlichen Vorstellung gaben und damit eine strukturierte postoperative Nachsorge sowie die frühzeitige Erkennung potenzieller Komplikationen im Sinne des mPOM-Konzeptes unterstützten.

Für das Behandlungsteam stand ein Dashboard zur Verfügung, das eine Übersicht über individuelle Fortschritte und patientenseitig eingegebene Informationen bot ( Abb. 1 **f** ). Das Monitoring erfolgte weitgehend automatisiert, um eine zusätzliche Arbeitsbelastung des Behandlungsteams zu vermeiden.

### Technische Umsetzung

Die technische Entwicklung und Implementierung der Patienten-App erfolgten in Kooperation mit dem niederländischen Digital-Health-Unternehmen Luscii Healthtech B. V., dessen Anwendung „Luscii“ als CE-zertifiziertes Medizinprodukt der Klasse IIa zugelassen ist. Die Nutzung erfolgte im Rahmen einer nicht interventionellen Beobachtungsstudie ohne Einfluss auf Diagnostik oder Therapie, sodass kein gesondertes Ethikvotum erforderlich war.

Alle Patientinnen und Patienten wurden vor Installation der App mündlich und schriftlich über Zweck, Ablauf, Datenschutz, Datenverarbeitung und Freiwilligkeit der Teilnahme informiert. Grundlage hierfür war ein gemeinsam mit dem Datenschutzbeauftragten des Universitätsklinikums Bonn erstellter und freigegebener Informations- und Einwilligungsbogen, den alle Teilnehmenden schriftlich bestätigten. Die Teilnahme war freiwillig, jederzeit widerrufbar und unabhängig von der klinischen Behandlung.

Der Zugang zur App erfolgte personalisiert durch Einladung und Freischaltung über eine Study Nurse sowie über einen passwortgeschützten Login. Die Anwendung war auf mobilen Endgeräten (iOS und Android) nutzbar. Die Datenverarbeitung erfolgte DSGVO-konform, ausschließlich auf Servern innerhalb der Europäischen Union, und sämtliche Übertragungen waren verschlüsselt.

### Fragebogen und Endpunkte

Zur Evaluation der Patienten-App beantworteten alle Patientinnen und Patienten 2 Fragebogen. Zum einen wurde der validierte GER-MAUQ, die deutsche Version des mHealth App Usability Questionnaire, eingesetzt 25 . Dieser erfasst standardisiert die Gebrauchstauglichkeit mobiler Gesundheitsanwendungen aus Patientensicht und bewertet Benutzerfreundlichkeit, Informationsqualität, Interaktion und subjektive Zufriedenheit auf einer 7-stufigen Likert-Skala. Ergänzend wurde ein projektspezifischer Fragebogen verwendet, der zusätzliche Items zur Nützlichkeit und Unterstützung, zu spezifischen Funktionen, emotionalen Aspekten und zur Gesamtbewertung enthielt. Auch diese Fragen wurden auf einer 7-stufigen Likert-Skala beantwortet. Beide Fragebogen wurden 30 Tage nach der Operation ausgefüllt.

Der primäre Endpunkt dieser Pilotstudie war die Akzeptanz und Benutzerfreundlichkeit der App. Sekundäre Endpunkte umfassten Nutzungserfahrung, Selbstmanagement-Aspekte, wahrgenommene Unterstützung sowie die Gesamtzufriedenheit.

### Statistische Analyse

Alle statistischen Analysen wurden mit GraphPad Prism, Version 10.4 für Mac (GraphPad Software, San Diego, CA, USA), durchgeführt. Die Basischarakteristika der Kohorte wurden für kontinuierliche Variablen als Mittelwerte und Standardabweichungen angegeben.

Aufgrund der geringen Fallzahl und des explorativen Studiendesigns erfolgte eine rein deskriptive Analyse der Fragebogendaten. Für die Likert-Skalen wurden sowohl die prozentuale Verteilung der 7 Antwortkategorien als auch die Mittelwerte der Items dargestellt. Ein p-Wert < 0,05 wurde als statistisch signifikant definiert.

## Ergebnisse

### Patientencharakteristika der Studienkohorte

In die Analyse wurden 14 Patientinnen und Patienten eingeschlossen, die sich zwischen Juni und Oktober 2025 einer elektiven kolorektalen Operation am Universitätsklinikum Bonn unterzogen und perioperativ die Patienten-App nutzten ( Tab. 1 ). Das mittlere Alter betrug 51,4 Jahre; 6 Teilnehmende (42,9%) waren männlich, 8 (57,1%) weiblich. Bei jeweils 7 Personen lag eine benigne Grunderkrankung bzw. ein kolorektales Karzinom vor. Die ASA-Klassifikation verteilte sich auf ASA II (n = 9), ASA III (n = 3) und ASA IV (n = 2). Die durchgeführten Eingriffe umfassten Reanastomosierungen eines bestehenden endständigen Anus praeter (n = 4), roboterassistierte Sigmaresektionen (n = 3), rechtsseitige Hemikolektomien (n = 2), roboterassistierte tiefe anteriore Rektumresektionen (n = 4) sowie eine abdominoperineale Pouch-Exstirpation (n = 1).

**Table:** **Tab. 1** Demografische und klinische Charakteristika der 14 Patientinnen und Patienten mit elektiver kolorektaler Operation am Universitätsklinikum Bonn unter perioperativer Nutzung der Patienten-App.

	Patienten (%)
n	14
**Geschlecht (%)**	
männlich	6 (42,86)
weiblich	8 (57,14)
**Alter zum Zeitpunkt der Operation (Jahre)**	
Mittelwert (SEM)	51,43 (± 4,4)
**Diagnose**	
benigne (%)	7 (50,00)
maligne (%)	7 (50,00)
**ASA-Klassifikation**	
I	0
II	9
III	3
IV	2
**Operation**	
a.–p Reanastomosierung eines endständigen Enterostomas	4
roboterassistierte Sigmaresektion	3
offene/laparoskopische Hemikolektomie rechts	2
roboterassistierte tiefe anteriore Rektumresektion	4
abdominoperineale Pouch-Exstirpation	1

## Usability und Nutzererfahrung

**Abb. 2:**
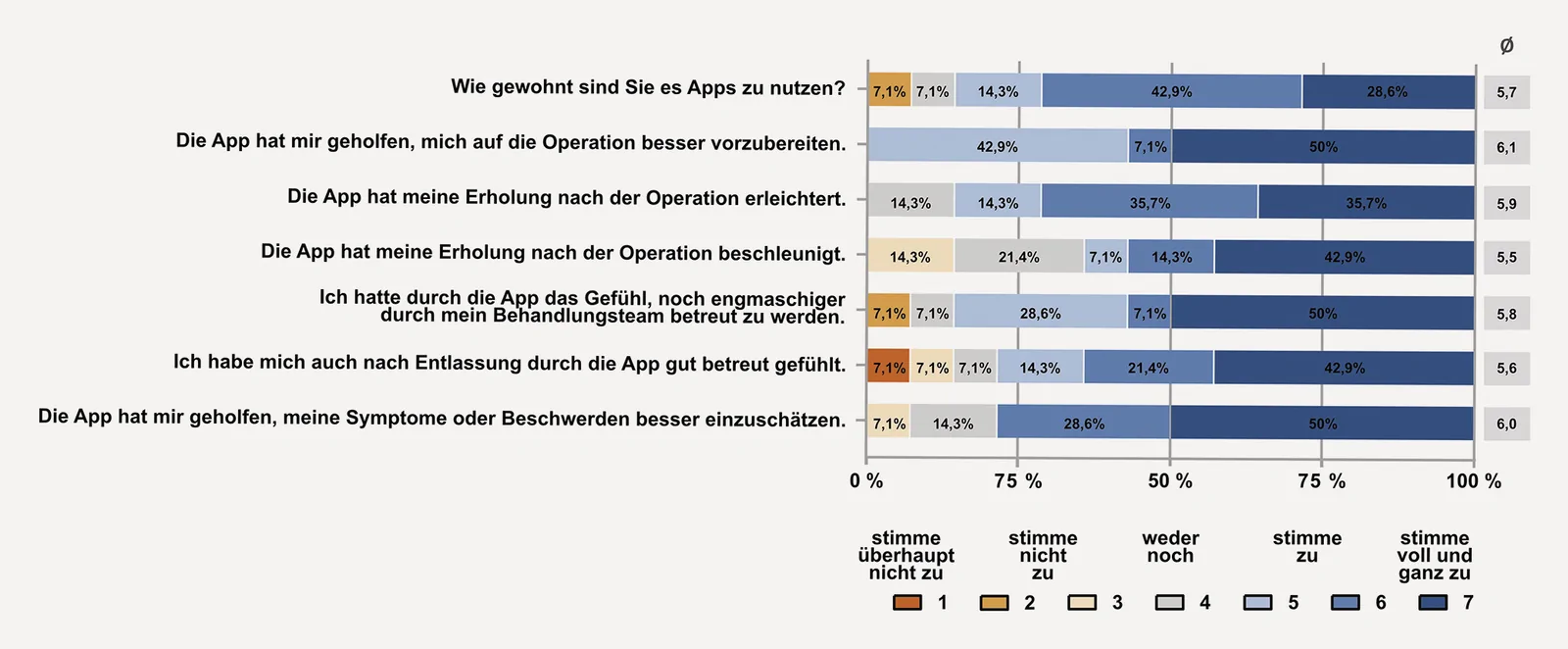
Bewertung der Usability und Nutzererfahrung der Patienten-App anhand einer 7-stufigen Likert-Skala. Dargestellt sind die prozentualen Anteile der Patientinnen und Patienten je Antwortkategorie pro Frage-Item (horizontale Balken). Rechts der Balken ist jeweils der arithmetische Mittelwert der Bewertung für das entsprechende Item angegeben.

Die Befragung aller 14 Patientinnen und Patienten zeigte eine insgesamt hohe Vertrautheit im Umgang mit mobilen Anwendungen ( Abb. 2 ). Zehn Teilnehmende bestätigten eine durchweg intuitive Bedienbarkeit sowie eine einfache Erlernbarkeit der App-Funktionen ( **Abb. S1** ). Auch funktionale Aspekte wie eine konsistente Navigation zwischen einzelnen Modulen und die Möglichkeit zur Korrektur eingegebener Angaben wurden überwiegend positiv bewertet.

Hinsichtlich Layouts, Strukturierung der Inhalte und Transparenz des individuellen Fortschritts berichteten die Patientinnen und Patienten eine hohe Zufriedenheit. Der zeitliche Aufwand wurde von 86% als angemessen empfunden, und die Mehrheit bestätigte eine stabile Nutzbarkeit auch bei eingeschränkter Internetverbindung.

Bezogen auf die Zielsetzung der Prähabilitation gaben alle Teilnehmenden an, in der präoperativen Phase durch die App unterstützt worden zu sein; 7 Personen äußerten hierbei maximale Zustimmung ( Abb. 2 ).

Etwa 85% empfanden auch die postoperative Erholung als erleichtert. Zudem berichteten 86% eine engmaschigere Betreuung durch das Behandlungsteam, und rund 80% fühlten sich auch im poststationären Verlauf gut begleitet.

Hinsichtlich der Selbsteinschätzung von Symptomen und Beschwerden gaben 11 Patientinnen und Patienten an, Symptome und Warnzeichen mithilfe der App besser einschätzen zu können; 2 äußerten sich neutral und eine Person eher kritisch. Die Unterstützung des Selbstmanagements der eigenen Gesundheit wurde von 9 Personen positiv bewertet, während 3 eine neutrale und 2 eine zurückhaltende Einschätzung abgaben ( Abb. 2 ); ergänzende GER-MAUQ-Ergebnisse in **Abb. S1** ).

## Bewertung der App-Funktionen

**Abb. 3:**
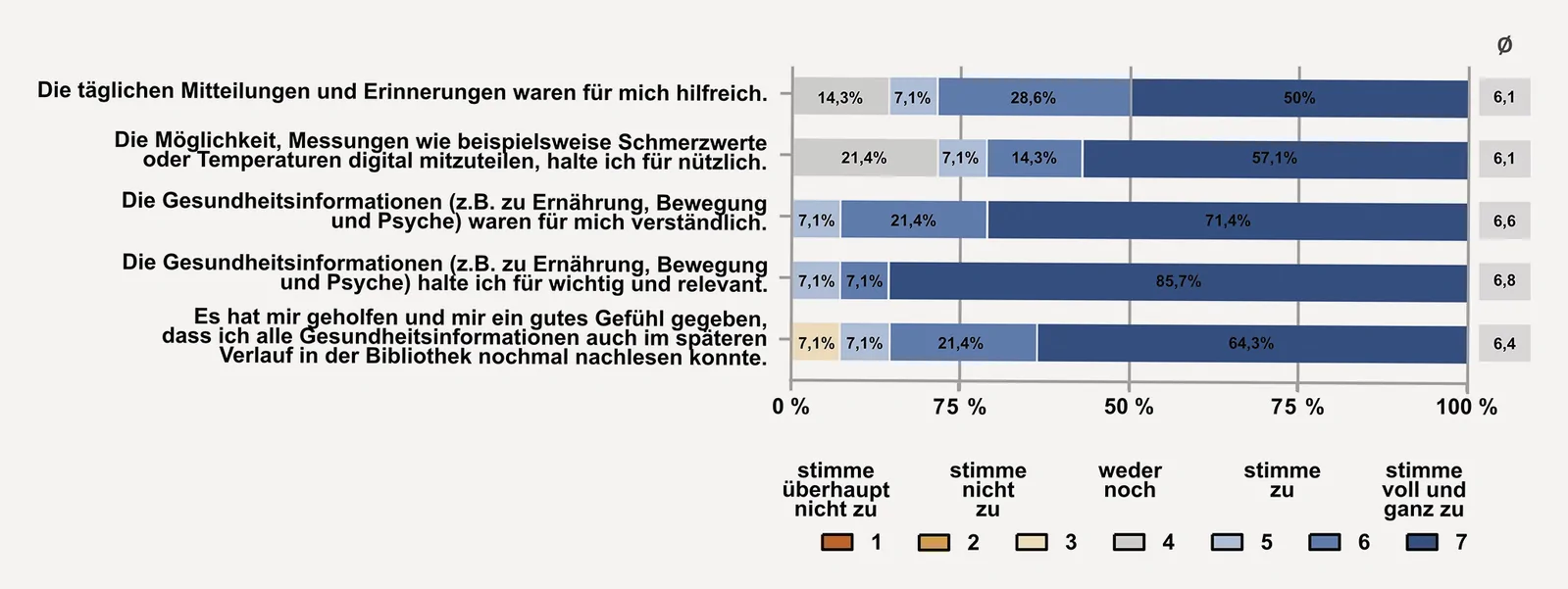
Bewertung spezifischer App-Funktionen anhand einer 7-stufigen Likert-Skala. Die Balken zeigen die prozentualen Anteile der Patientinnen und Patienten je Antwortkategorie. Rechts der Balken ist jeweils der arithmetische Mittelwert der Bewertung pro Item angegeben.

Die funktionalen Elemente der Patienten-App wurden insgesamt sehr positiv bewertet. Die täglichen Mitteilungen und Erinnerungsfunktionen erhielten von 50% der Befragten die Höchstwertung, weitere rund 30% vergaben 6 von 7 Punkten ( Abb. 3 ). Auch die Möglichkeit zur digitalen Übermittlung klinisch relevanter Parameter wie Schmerzintensität oder Körpertemperatur wurde von 60% der Patientinnen und Patienten mit der höchsten Punktzahl bewertet.

Die bereitgestellten Gesundheitsinformationen zu Ernährung, Bewegung und psychischer Gesundheit wurden durchweg als verständlich und klinisch relevant eingeschätzt. Über 90% bewerteten zudem den dauerhaften Zugriff auf diese Inhalte über die integrierte Wissensbibliothek als hilfreich und sicherheitsstiftend.

Darüber hinaus gaben 71% der Teilnehmenden an, dass die App den Zugang zu Gesundheitsleistungen, wie beispielsweise Arztbesuchen, erleichtert habe ( **Abb. S1** ). Elf von 14 Teilnehmenden bestätigten, dass die App ihren Erwartungen an Funktionsumfang und Nutzungsmöglichkeiten entsprochen habe. Sieben Teilnehmende stimmten uneingeschränkt zu, dass die App einen geeigneten Zugang zu Informationsmaterial, Aktivitätsverfolgung und Selbsteinschätzung bietet.

## Emotionale und wahrnehmungsbezogene Aspekte der App-Nutzung

**Abb. 4:**
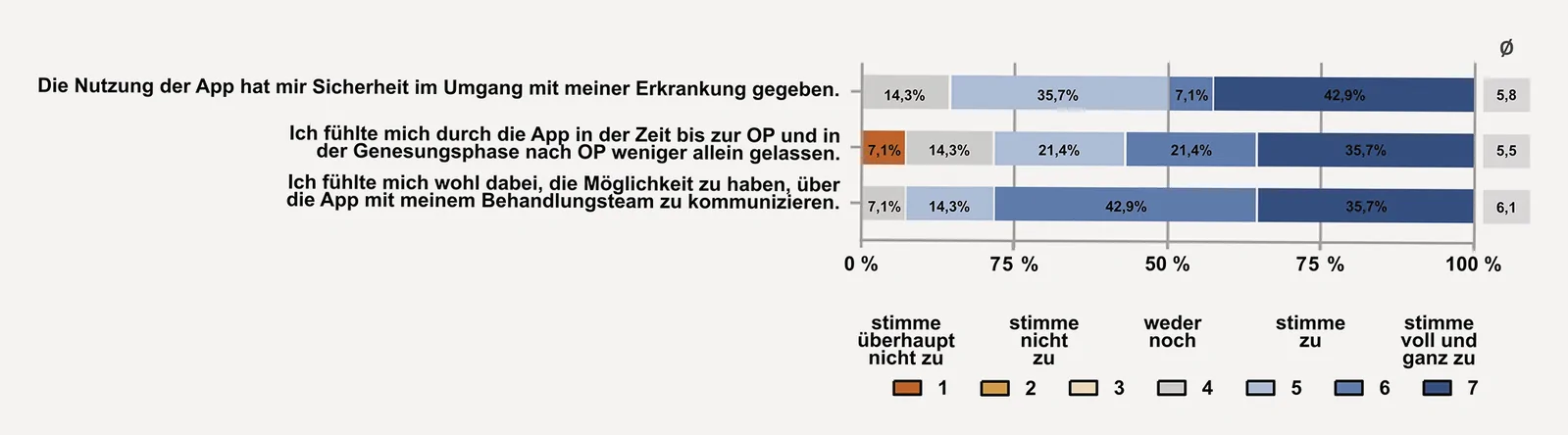
Bewertung emotionaler und wahrnehmungsbezogener Aspekte der App-Nutzung auf einer 7-stufigen Likert-Skala. Die Balkendiagramme zeigen die prozentualen Anteile der Patientinnen und Patienten je Antwortkategorie; rechts neben den Balken ist jeweils der arithmetische Mittelwert des entsprechenden Items ausgewiesen.

In Bezug auf die emotionale Dimension der App-Nutzung berichteten 6 von 14 Patientinnen und Patienten eine maximale Zustimmung dafür, dass die Anwendung ein erhöhtes Gefühl von Sicherheit im Umgang mit der Erkrankung vermittelt habe. Weitere 6 Personen vergaben 5 bzw. 6 von 7 Punkten ( Abb. 4 ).

Rund 80% der Befragten gaben zudem an, sich durch die App sowohl präoperativ als auch während der postoperativen Genesung weniger alleingelassen gefühlt zu haben. Eine Person widersprach dieser Einschätzung, 2 verhielten sich neutral.

Auch die Kommunikationsfunktion mit dem Behandlungsteam wurde positiv bewertet: 36% der Teilnehmenden vergaben die Höchstwertung, weitere 43% 6 von 7 Punkten. Zwei Personen bewerteten diesen Aspekt mit 5 Punkten, eine Person neutral.

Darüber hinaus fühlten sich 11 der 14 Patientinnen und Patienten wohl dabei, die App auch in sozialen Situationen, beispielsweise im Beisein anderer Menschen im Wartezimmer oder Fitnessstudio, zu nutzen, was auf eine geringe soziale Hemmschwelle und eine hohe Alltagstauglichkeit der Anwendung hinweist (Abb. S1).

### Gesamtbewertung

**Abb. 5:**
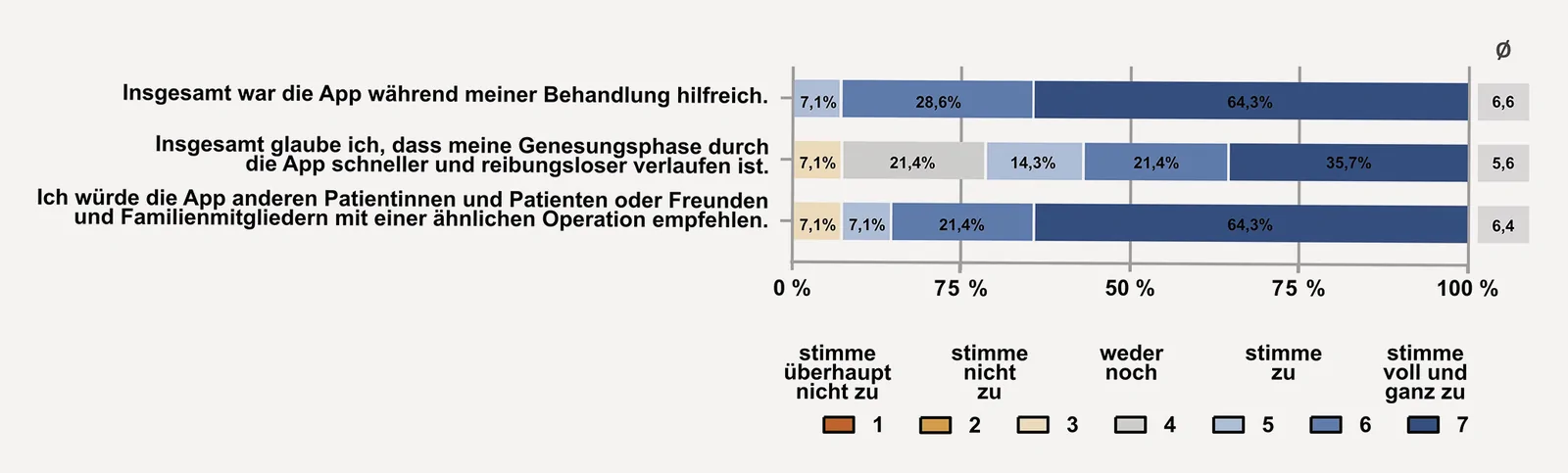
Gesamtbewertung der Patienten-App auf einer 7-stufigen Likert-Skala. Die Balkendiagramme zeigen die prozentualen Anteile der Patientinnen und Patienten je Antwortkategorie; der arithmetische Mittelwert des jeweiligen Items ist rechts neben den Balken ausgewiesen.

In der Gesamtbewertung stuften knapp 65% der Patientinnen und Patienten die App während der Behandlung als hilfreich ein; weitere rund 30% vergaben 6 von 7 Punkten ( Abb. 5 ). Zudem waren 71% der Auffassung, dass die postoperative Genesungsphase durch die Nutzung der App schneller und reibungsloser verlaufen sei.

Zehn der 14 Teilnehmenden würden die App erneut nutzen und äußerten dabei maximale Zufriedenheit mit der Anwendung. Entsprechend hoch war auch die Weiterempfehlungsbereitschaft: Neun Personen würden die App uneingeschränkt weiterempfehlen, 3 vergaben 6 von 7 Punkten, eine Person 5 Punkte und eine Person 3 Punkte.

## Diskussion

Die vorliegenden Ergebnisse zeigen, dass eine umfassende Patienten-App, die alle wesentlichen Inhalte des perioperativen Patientenpfades, einschließlich Prähabilitation, Fast Track/mPOM-Elementen und postoperativem Monitoring, integriert, in der Kolorektalchirurgie sowohl gut implementierbar ist als auch eine hohe Akzeptanz findet. Dabei wurden zentrale Bestandteile etablierter ERAS- und mPOM-Konzepte digital abgebildet und in den klinischen Alltag übertragen. Hierzu gehörten insbesondere die strukturierte präoperative Patientenedukation, prähabilitative Maßnahmen mit Fokus auf Ernährung, körperliche Aktivität und Nikotin- bzw. Alkoholkarenz, perioperative Erinnerungen zur frühzeitigen Mobilisation und enteralen Ernährung sowie standardisierte postoperative Symptomabfragen zur frühzeitigen Erkennung potenzieller Komplikationen. Durch diese digitale Umsetzung wurden evidenzbasierte Maßnahmen nicht nur vermittelt, sondern zeitlich strukturiert begleitet und für die Patientinnen und Patienten kontinuierlich nachvollziehbar gemacht. Alle Patienten und Patientinnen nutzen die Anwendung bis zum Abschluss der Behandlung regelmäßig. Dies unterstreicht gemeinsam mit den sehr hohen Zufriedenheitswerten den praktischen Nutzen und die Relevanz digitaler Begleitangebote in einer chirurgischen Patientengruppe.

Im deutschsprachigen Raum existieren bisher keine Daten zur systematischen Implementierung einer perioperativen Patienten-App in der Kolorektalchirurgie, sodass unsere Untersuchung erstmals die Machbarkeit und Akzeptanz eines mPOM-basierten digitalen Versorgungspfades in der klinischen Routine belegt.

Die Ergebnisse stehen im Einklang mit internationalen Beobachtungen, insbesondere aus den Niederlanden und Kanada, in denen mobile Anwendungen bereits erfolgreich zur Prähabilitation, Informationsvermittlung und postoperativen Verlaufskontrolle eingesetzt wurden. Talen et al. 19 berichteten von einer Verbleiberate von 80% und einem Zufriedenheits-Score von 5,4 auf einer Likert-Skala von 1 bis 7, während in der Studie von Keng et al. 26 92% der Patienten und Patientinnen die Zufriedenheit mit der mobilen App als hoch bis sehr hoch bewerteten. In größeren Studienkohorten sank die Adhärenz jedoch auf unter 60% ab 27 28 29 , was die Relevanz einer hohen Usability betont.

In der GER-MAUQ-Auswertung bestätigte sich die Benutzerfreundlichkeit unserer App: Bedienbarkeit, Verständlichkeit und Navigation wurden überwiegend positiv bewertet. Dies deckt sich mit internationalen mHealth-Studien, in denen chirurgische Patienten-Apps ebenfalls durch hohe Usability-Scores auffallen 19 30 31 und ist deshalb bedeutsam, weil eine intuitive Nutzung entscheidend für die nachhaltige Integration digitaler Anwendungen in das perioperative Setting ist. Die hohe Alltagstauglichkeit – die App wurde problemlos auch in sozialen Situationen genutzt – weist zudem auf eine geringe Nutzungshürde hin.

Ein zentrales Ergebnis ist die deutliche Unterstützung der Patientinnen und Patienten in der präoperativen Phase. Die Prähabilitation ist in Deutschland bislang weder strukturell implementiert noch finanziert, sodass die Hoffnung hier explizit auf digitale Lösungen gelegt wird 4 6 .

Auch wenn unsere Fallzahl keine Aussagen zu Outcome-Parametern erlaubt, unterstreicht die Literatur das Potenzial strukturierter Prähabilitation 32 33 . Studien zeigen, dass durch strukturierte Prähabilitationsprogramme die Komplikationsrate um 41–50% gesenkt 34 35 , die postoperative Erholung verbessert 36 und Kostenersparnisse von über 20% erzielt werden können 37 . Neuere Arbeiten zeigen zudem, dass Wearables die Effektivität prähabilitativer Maßnahmen weiter verstärken können 38 39 .

Eine Besonderheit unseres digitalen Prähabilitationskonzeptes ist zudem die Erweiterung der klassischen Wirkungsbereiche Physiotherapie und Ernährungsberatung um den Themenkomplex Psychologie und Stressreduktion. Hierzu trug insbesondere die Bereitstellung angeleiteter Entspannungsübungen wie Bodyscan und Progressive Muskelrelaxation bei, die von den Patientinnen und Patienten aktiv genutzt wurden. Diese Elemente sind in Prähabilitationsstudien bislang unterrepräsentiert 40 , obwohl sie perioperative Angst und depressive Symptome nachweislich reduzieren können 41 . Zwar gibt es Patienten-Apps, in denen Depression und Angst abgefragt werden 42 , psychologische Interventionen wie Entspannungsübungen wurden jedoch bisher nur selten aktiv vermittelt 39 43 44 , sodass unser Konzept hier eine inhaltliche Erweiterung darstellt.

Neben den edukativen Inhalten zeigte sich auch ein positiver Einfluss auf das Verhalten der Patientinnen und Patienten: Die App stärkte die aktive Rolle im Sinne des mPOM-Konzeptes. Mehrere Rückmeldungen zeigten, dass die Nutzenden durch die vermittelten Inhalte zu einem aufmerksameren und selbstbestimmteren Umgang mit ihrem Genesungsprozess angeleitet wurden – etwa indem sie nachhakten, wenn erwartete Elemente wie frühzeitige Mobilisation oder eine feste Koststeigerung nicht erfolgten. Zudem fühlten sich knapp 80% im perioperativen Verlauf weniger allein gelassen, was auf eine gestärkte Bindung zum Behandlungsteam und einen Zugewinn an Selbstwirksamkeit hinweist. Diese Befunde sind konsistent mit der mHealth-Forschung, die eine Verbesserung von Selbstmanagement und Symptomkompetenz durch digitale Interventionen beschreibt 45 46 .

Auch postoperative Aspekte, insbesondere das individuelle Erleben des Genesungsprozesses, wurden überwiegend positiv bewertet; rund 85% der Befragten berichteten eine erleichterte Erholung. Dies legt nahe, dass digitale Begleitangebote nicht nur zur Wissensvermittlung beitragen, sondern auch das subjektive Sicherheitsempfinden im poststationären Verlauf stärken können. Damit stehen unsere Ergebnisse in Einklang mit der Studie von Pooni et al. 10 , in der Zufriedenheit und Wohlbefinden nach Entlassung durch eine App signifikant gesteigert werden konnten.

Ein weiterer wesentlicher Vorteil liegt in der strukturierten Erfassung prä- und postoperativer Warnsymptome. Regelmäßige Abfragen zu Schmerzen, Fieber oder Wundveränderungen können nicht nur das subjektive Sicherheitsempfinden stärken, sondern auch zu einer früheren und zuverlässigeren Erkennung klinisch relevanter Symptome beitragen und damit die Patientensicherheit erhöhen. Auch die regelmäßige Erfassung der präoperativen Gewichtsentwicklung kann dazu beitragen, unbeabsichtigte Gewichtsverluste als Hinweis auf Frailty oder Malnutrition frühzeitig zu erkennen und damit potenziell relevante Risikokonstellationen schneller zu adressieren. In der vorliegenden Pilotkohorte war die präoperative Phase jedoch häufig relativ kurz, sodass Veränderungen des Körpergewichts nur eingeschränkt longitudinal beurteilt werden konnten. Dies ist typisch für viele elektive kolorektalchirurgische Eingriffe, bei denen zwischen Operationsindikation und Eingriff häufig nur ein begrenzter Zeitraum liegt. Ein besonderes Potenzial des digitalen Gewichtsmonitorings ergibt sich jedoch bei Patientinnen und Patienten mit längeren präoperativen Therapiephasen, etwa im Rahmen einer neoadjuvanten Therapie beim Rektumkarzinom. In diesen Situationen kann eine kontinuierliche Gewichtsdokumentation helfen, nutritive Risiken frühzeitig zu erkennen und gezielt zu adressieren. Darüber hinaus ermöglicht die Erfassung eines präoperativen Ausgangswertes auch eine bessere Beurteilung postoperativer Gewichtsveränderungen, beispielsweise bei Patientinnen und Patienten mit Stomaanlage. Mit zunehmender Integration neoadjuvanter Konzepte auch beim Kolonkarzinom, wie etwa im Rahmen der FOxTROT-Studie beschrieben 47 , könnte die Bedeutung eines strukturierten präoperativen Gewichtsmonitorings künftig weiter zunehmen. Die digitale Erfassung sowie die Ableitung entsprechender Empfehlungen erfolgte dabei ohne zusätzliche manuelle Dateneingabe seitens des Behandlungsteams, sodass ein engmaschigeres Monitoring ohne zusätzliche Ressourcenbindung möglich wurde. Ein Aspekt, der auch in anderen Studien als entscheidend für die langfristige Implementierbarkeit beschrieben wird 19 . Perspektivisch könnte dies sogar eine frühere Entlassung mit digital unterstützter Überwachung zu Hause ermöglichen. Dieser Ansatz eines „Hospital-at-Home“-Modells war nicht Ziel unserer Pilotstudie, stellt jedoch den logischen nächsten Entwicklungsschritt dar. Internationale Studien – insbesondere aus Nordamerika – zeigen, dass patientenbegleitende Apps sogar für tagesgleiche Entlassungen eingesetzt werden können 48 und dabei ökonomisch hoch relevante Parameter wie Notfallvorstellungen, Wiederaufnahmerate und Krankenhausverweildauer signifikant verbessern 9 11 26 49 50 .

Angesichts der zunehmenden Ressourcenknappheit im stationären Bereich 51 könnte eine solche digitale Nachsorgestruktur auch in der Kolorektalchirurgie substanzielle Effekte auf Sicherheit, Versorgungsqualität und Wirtschaftlichkeit entfalten. Darüber hinaus lässt sich das digitale Monitoring durch den Einsatz von Wearables deutlich erweitern. Sensorbasierte Daten zu Aktivität, Mobilität und Vitalparametern könnten eine noch engmaschigere und objektivere Verlaufsbeurteilung im häuslichen Umfeld prä- und postoperativ ermöglichen 52 53 54 55 56 57 und – insbesondere im Bereich Mobilität und Bewegung – sogar zu verbesserten postoperativen Ergebnissen beitragen 58 59 .

Neben den patientenspezifischen Effekten zeigte sich auch ein klarer organisatorischer Nutzen.

Die bereitgestellten Gesundheitsinformationen – von Aufklärungsvideos bis zur Packliste – wurden als besonders hilfreich und sicherheitsstiftend bewertet, insbesondere durch den permanenten Zugriff über die integrierte Wissensbibliothek.

Durch standardisierte digitale Informationen kann die persönliche Aufklärung und Betreuung sinnvoll ergänzt und das pflegerische und ärztliche Personal gleichzeitig entlastet werden, da die Patienten bereits vorinformiert sind und viele vermeintliche Banalitäten („Wie funktioniert der Fernseher?“) im Vorfeld geklärt werden. Als besonders hilfreich wurden die Aufgaben- und Erinnerungsfunktionen genannt, die strukturierend wirken und im Verlauf wiederholt zu gesundheitsrelevanten Maßnahmen motivieren. Zudem ermöglicht die App die digitale Erhebung zertifizierungsrelevanter Patientenfragebogen, etwa des Distress-Thermometers oder zur Ermittlung des Risikos für eine erbliche Form von Darmkrebs. Dies führt zu einer weiteren Entlastung des medizinischen Teams und gleichzeitig zu höheren Rücklaufquoten, was in Rahmen von Zertifizierungen einen strukturellen Vorteil darstellt. Ein zusätzlicher Nutzen bestand darin, dass 71% der Teilnehmenden angaben, dass ihnen die App den Zugang zu Gesundheitsleistungen – etwa Terminorganisation oder Nachsorgekontakte – erleichtert habe.

Die kontinuierliche digitale Begleitung stärkt zudem die Bindung der Patientinnen und Patienten an das Behandlungsteam und trägt zu einem frühzeitigen Vertrauensaufbau bei. Dies fördert die Adhärenz zu Prähabilitations- und mPOM-Maßnahmen, reduziert Unsicherheiten im perioperativen Verlauf und unterstützt eine aktivere Mitgestaltung des Genesungsprozesses. Damit kann eine digital unterstützte Patientenführung nicht nur die wahrgenommene Versorgungsqualität verbessern, sondern langfristig auch zur Attraktivität eines Behandlungszentrums beitragen – ein Aspekt, der im Wettbewerb um Patientinnen und Patienten zunehmend an Bedeutung gewinnt.

Trotz der positiven Ergebnisse müssen die Limitationen dieser Pilotstudie berücksichtigt werden. Es handelt sich um eine kleine, selektierte Kohorte, deren Umfang noch keine belastbaren Aussagen zu klinischen Endpunkten oder Outcome-Analysen im Vergleich zu einer Kontrollgruppe erlaubt.

Das Design als Single-Center-Studie limitiert zudem die Übertragbarkeit. Da in dieser frühen Phase bewusst eher digital affine Patienten und Patientinnen eingeschlossen wurden, ist die Generalisierbarkeit eingeschränkt. Dies entspricht Beobachtungen aus anderen mHealth-Studien, die eine altersabhängige digitale Nutzungslücke („Digital Divide“) zwischen digital affinen und vulnerableren Patientengruppen beschreiben 19 . Künftige Studien sollten daher größere und heterogenere Kohorten einbeziehen, die Vergleichbarkeit mit konventionellen Versorgungspfaden untersuchen und sowohl klinische Resultate als auch gesundheitsökonomische Effekte berücksichtigen.

## Schlussfolgerung

Insgesamt zeigt die vorliegende Pilotstudie, dass eine umfassende, multimodale digitale Patientenführung im gesamten perioperativen Verlauf der Kolorektalchirurgie hohe Akzeptanz findet und eine sinnvolle Ergänzung etablierter Behandlungskonzepte darstellt. Die sehr positiven Rückmeldungen zu Benutzerfreundlichkeit, prähabilitativer Unterstützung und postoperativem Monitoring unterstreichen das Potenzial digitaler Anwendungen, Versorgungsprozesse strukturiert abzubilden, die Patientensicherheit zu erhöhen und die selbstbestimmte Mitgestaltung des Genesungsprozesses zu fördern.

Gleichzeitig verdeutlichen die Ergebnisse, dass digitale Tools einen organisatorischen Mehrwert bieten können, etwa durch standardisierte Informationen, Erinnerungsfunktionen und die strukturierte Erfassung klinisch relevanter Parameter.

Um den langfristigen Nutzen, die Auswirkungen auf klinische Outcomes und die Einbindung in standardisierte mPOM-Pfade verlässlich beurteilen zu können, sind prospektive Studien mit größeren und heterogeneren Kohorten erforderlich. Die Kombination aus edukativen Inhalten, motivationsfördernden Elementen und systematischem Monitoring stellt dabei einen vielversprechenden Ansatz zur Qualitäts- und Sicherheitssteigerung in der chirurgischen Versorgung dar.

## Zusatzmaterial

Supplementary Material Zusatzmaterial
