# Supplementary material for: Evaluation of Multimodal Perioperative Care Pathway Supported by a Patient-facing Mobile App in Colorectal Surgery - First Clinical Experience and Patient Satisfaction
Source: Zentralbl Chir. 2026 Jun 8;151(4):353–63. [Article in German] doi: 10.1055/a-2862-3085 (PMC13489686; doi:10.1055/a-2862-3085)
Supplement: Supplementary file 1 [file 10-1055-a-2862-3085_28852004.pdf]

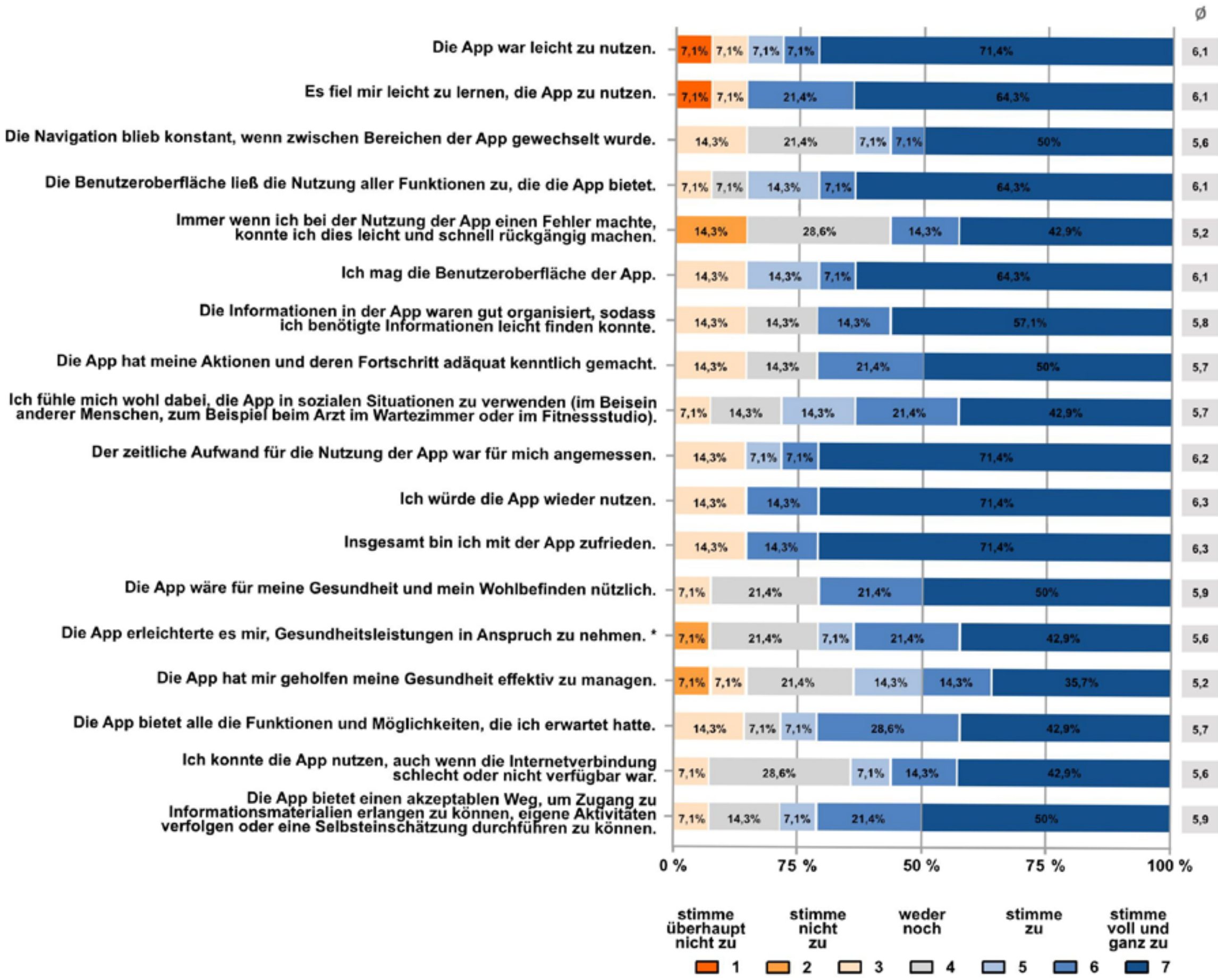

Abb. S1 Auswertung des GER-MAUQ-Fragebogens anhand einer 7-stufigen Likert-Skala. Die Balkendiagramme zeigen die prozentualen Anteile der Patientinnen und Patienten je Antwortkategorie; der arithmetische Mittelwert des jeweiligen Items ist rechts neben den Balken dargestellt.
